# Avian Malaria Parasites Modulate Gut Microbiome Assembly in Canaries

**DOI:** 10.3390/microorganisms11030563

**Published:** 2023-02-23

**Authors:** Justė Aželytė, Alejandra Wu-Chuang, Apolline Maitre, Rita Žiegytė, Lourdes Mateos-Hernández, Dasiel Obregón, Vaidas Palinauskas, Alejandro Cabezas-Cruz

**Affiliations:** 1 Nature Research Centre, Akademijos 2, LT-09412 Vilnius, Lithuania; 2 ANSES, INRAE, Ecole Nationale Vétérinaire d’Alfort, UMR BIPAR, Laboratoire de Santé Animale, F-94700 Maisons-Alfort, France; 3 INRAE, UR 0045 Laboratoire de Recherches Sur Le Développement de L’Elevage (SELMET-LRDE), F-20250 Corte, France; 4 EA 7310, Laboratoire de Virologie, Université de Corse, F-20250 Corte, France; 5 School of Environmental Sciences, University of Guelph, Guelph, ON N1G 2W1, Canada

**Keywords:** avian malaria, microbiome, parasite-microbiota interactions

## Abstract

Rodent and human malaria parasites cause dysbiosis in the host gut microbiome, but whether *Plasmodium* species affecting birds cause dysbiosis in their hosts is currently unknown. Here we used a model of avian malaria infection to test whether parasite infection modulates the bird microbiome. To this aim, bird fecal microbiomes were characterized at different time points after infection of canaries with the avian malaria parasite *Plasmodium homocircumflexum*. Avian malaria caused no significant changes in the alpha and beta diversity of the microbiome in infected birds. In contrast, we discovered changes in the composition and abundance of several taxa. Co-occurrence networks were used to characterize the assembly of the microbiome and trajectories of microbiome structural states progression were found to be different between infected and uninfected birds. Prediction of functional profiles in bacterial communities using PICRUSt2 showed infection by *P. homocircumflexum* to be associated with the presence of specific degradation and biosynthesis metabolic pathways, which were not found in healthy birds. Some of the metabolic pathways with decreased abundance in the infected group had significant increase in the later stage of infection. The results showed that avian malaria parasites affect bacterial community assembly in the host gut microbiome. Microbiome modulation by malaria parasites could have deleterious consequences for the host bird. Knowing the intricacies of bird-malaria-microbiota interactions may prove helpful in determining key microbial players and informing interventions to improve animal health.

## 1. Introduction

The scarcity of gut microbiome studies compared with other taxonomic groups underscores a gap in our understanding of host-microbiota interactions in metazoans [1]. Next-generation sequencing and microbiome analyses in different bird species [2] provide a significant contribution to our understanding of the diversity of microbial communities in the gut microbiota of metazoans and a first insight into the functional contribution of bird microbiome to host physiology [3]. Studies on the identification of extrinsic and intrinsic factors affecting gut microbiome composition revealed that host genetics is among the most important factors accounting for differences in the composition of gut microbiome in wild birds [4]. The local habitat of the birds was also shown to have a large influence on gut microbiome composition in Arctic-breeding shorebirds [5], and Brown-headed Cowbirds [6]. The ecological implications of microbiota diversity in wild birds remain to be explored [7].

In addition to host genetics and environmental factors, empirical evidence shows that parasite infection triggers changes in host microbiome [8,9,10]. For example, a recent study by Videvall et al. [11] showed differential bacterial abundance in the microbiome of uropygial gland of *Plasmodium*-infected house sparrows compared to uninfected birds. These results suggest that *Plasmodium* infection may affect the abundance, but also the composition and/or diversity of commensal bacteria associated with the host. This has been shown experimentally for murine malaria parasites [12,13,14]. By disrupting the microbiota-immune system homeostasis, malaria alters the profiles of mouse gut microbiome [12,13,14]. Two independent studies provided evidence that infection with rodent malaria parasites *Plasmodium yoelii* [15] and *Plasmodium berghei* [16] resulted in alterations in the gut microbiome profile. Similar results were reported for other apicomplexan parasites such as *Eimeria* spp. in chicken [10] and *Cryptosporidium parvum* in goat kids [8]. Mechanistically, deviation from immune homeostasis due to malaria infections may impact the host microbiome composition, and malaria-related changes in the composition of the microbiome may further alter the host immunity [14], which may result in different patterns of disease susceptibility and/or severity.

Based on morphological and mitochondrial genome features, more than 55 species of avian malaria parasites have been described [17,18]. Host specificity of malaria parasites varies from specialists infecting a single bird species to generalists infecting more than 300 distantly-related bird species [17,18,19]. *Plasmodium homocircumflexum* (COLL4) is known to infect around 19 different bird species belonging to 11 families (MalAvi v. 2.5.5) [18]. The distribution of this parasite in wildlife populations is not well known, however, the detection of *P. homocircumfelxum* in the European migratory birds, wintering in Africa, and resident birds of Africa and South America regions indicates its natural occurrence (MalAvi v. 2.5.5) [18]. Experimental infections of several bird species showed that this parasite can be highly virulent [20,21,22], however, the pathologies caused by *P. homocircumflexum* varies in different bird species, which might appear due to the adaptation of parasite gene expression in the avian hosts of different species [23]. The pathogenicity of *P. homocircumfelxum* is manifested by the development of severe parasitemia and/or exoerythrocytic stages in various organs leading to death of the host [21,22].

In this study, we tested whether experimental infection of canaries (*Serinus canaria domestica*) by the generalist avian malaria parasite *P. homocircumflexum* modulate the bird host gut microbiome from acute and chronic stages of the primary infection to the beginning of the latent stage. The results support that, as with murine and human malaria, avian malaria parasites influence the composition and structure of commensal bacteria populations in the host gut.

## 2. Material and Methods

### 2.1. Ethical Statement

All procedures were performed at Nature Research Centre in Vilnius, Lithuania, according to Lithuanian and International Guiding Principles for Biomedical Research Involving Animals (2012). Infection experiments were reviewed and approved by the Lithuanian State Food and Veterinary Service, Ref. No 2020/07/24-G2-84. The assessment of the animal health and all described procedures were implemented by trained professionals (under licenses 2012/02/06-No-208, 2016/01/29-No-344, and 2021/02/05-No-527).

### 2.2. Birds and Housing Conditions

One-year-old domestic canaries (*Serinus canaria domestica*) were kept in the same room of Nature Research Centre vivarium (License No. LT-61-13-003) under standard living conditions for birds. Experimental birds were housed in cages individually. The facilities were under a controlled temperature of 21 °C throughout the duration of the experiment. Standard food for canaries and water were provided *ad libitum*.

### 2.3. Experimental Design

**Figure 1:**
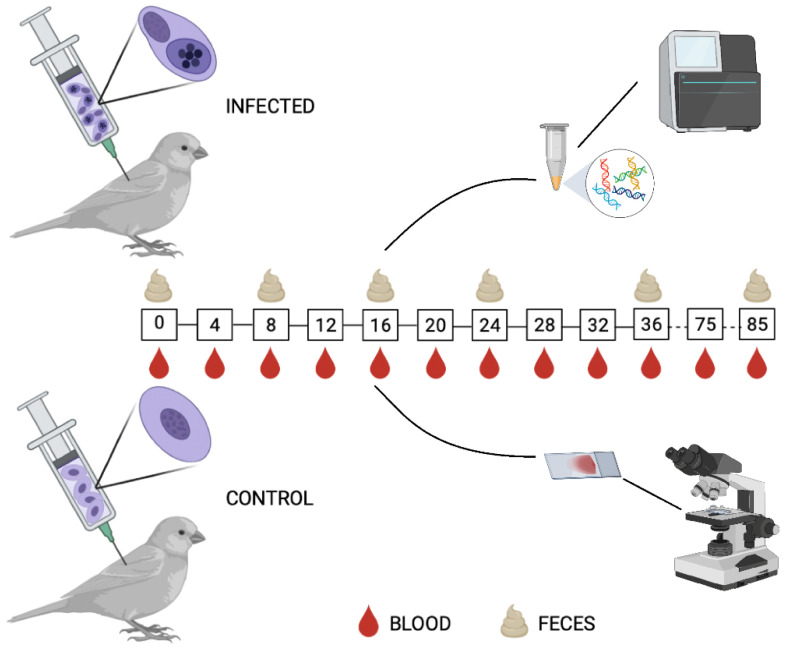
Experimental design. Canaries were inoculated with *P. homocircumflexum-*infected (*n* = 8) or uninfected (*n* = 8) donor blood. Blood and fecal samples were collected at different time points as indicated. Created with BioRender.com.

Canaries were used as a model for avian malaria infection with *P. homocircumflexum* parasite. Before the experimental procedures began, birds were randomly separated in ‘infected’ and ‘control’ groups (Figure 1). Birds in the control group received blood from uninfected bird donors. The infected group of birds received an inoculation of infectious blood with *P. homocircumflexum* parasites. Every four days post-infection (DPI) blood samples were collected and parasitemia was measured. Bird feces were collected and used for DNA extraction and microbiome analysis using bacterial 16S rRNA amplicon sequencing to test for an impact of malaria infection on bird gut microbiome. Experimental procedures are described below.

### 2.4. Thawing the Cryopreserved Avian Malaria Sample

The cryopreserved *P. homocircumflexum* strain COLL4 was thawed and used to infect donor canaries. Samples containing *P. homocircumflexum*-infected avian blood, cryopreserved in liquid nitrogen, were thawed as described by Dimitrov et al. [24]. Briefly, thawed samples were mixed with 12% NaCl (1/3 of thawed sample amount). After 5 min equilibration, one volume of 1.6% of NaCl was added followed by centrifugation at 1400 rpm for 5 min. After centrifugation, the supernatant was removed and 1.6% NaCl (1/3 of original sample) was added and centrifuged again. The same procedure was repeated three times with 0.9% NaCl solution. The final mixture was diluted with 0.9% NaCl and sub-inoculated into two donor canaries.

### 2.5. Experimental Infection of Birds

Birds from one group (*n* = 8) were experimentally infected with *P. homocircumflexum* using the protocol described by Palinauskas et al. [25]. Each experimental bird was sub-inoculated with a mixture (0.10 mL) of infected blood, 3.7% sodium citrate and 0.9% saline in proportion 4:1:5 into the pectoral muscles. Each bird received approximately 3 × 10^5^ of mature *P. homocircumflexum* meronts. The duration of experiment was 85 DPI. Parasitemia was examined every 4 DPI until 36 DPI by taking blood from the brachial vein as described in the section below. After 36 DPI, parasitemia was measured two more times at 75 and 85 DPI (Figure 1).

### 2.6. Blood Sample and Bird Feces Collection

Blood samples were taken from birds by puncturing brachial vein using heparinized microcapillaries. A small drop of blood was used to make two smears for microscopy to count erythrocytic stages in the blood (see below). Smears were air-dried, fixed with absolute methanol and stained as described by Valkiūnas et al. [26]. A fraction of blood (20–30 μL) was placed in SET-buffer for molecular analysis (PCR, see below) to confirm the lineage in recipient birds. The blood for smears and SET-buffer was collected at 0 DPI prior to the inoculation of infected and non-infected blood and every 4 DPI until 36 DPI, and on 75 and 85 DPI (Figure 1). Fresh feces were collected from each bird in sterile tubes at the same time as blood taking on 0, 8, 16, 24, 36, and 85 DPI. Samples were stored at −20 °C before genomic DNA extraction.

### 2.7. Microscopic Examination

We used an Olympus BX61 light microscope (Olympus, Tokyo, Japan) to examine blood smears and calculated the parasitemia as a percentage by actual counting of the number of parasites per 10,000 erythrocytes as described by Godfrey et al. [27].

### 2.8. DNA Extraction and PCR for P. homocircumflexum Identification

Total DNA for PCR analysis was extracted from the blood stored in SET-buffer using an ammonium acetate extraction protocol by Sambrook and Russel [28]. We performed a nested PCR following a protocol described by Hellgren et al. [29] to confirm *P. homocircumflexum* infection. For the first PCR we used the primers HaemNFI [5′-CATATATTAAGAGAAITATGGAG-3′] and HaemNR3 [5′-ATAGAAAGATAAGAAATACCATTC-3′] [29]. In the second PCR a fragmentt of mitochondrial *cyt b* gene (478 bp) was amplified using the primers HaemF [5′-ATGGTGCTTTCGATATATGCATG-3′] and HaemR2 [5′-GCATTATCTGGATGTGATAATGGT-3′] [30]. For PCR mix we used 12.5 μL of DreamTaq Master Mix (Thermo Fisher Scientific, Vilnius, Lithuania), 8.5 μL of nuclease-free water, 1 μL of each primer and 2 μL of template DNA (extracted DNA or products of first PCR). *Plasmodium homocircumflexum*-positive samples were determined by running 2 μL of second PCR product on 2% agarose gel. For parasite lineage confirmation, samples containing parasite DNA were sequenced from the 5′end using the HAEMF primer on an ABI PRISM TM 3100 capillary sequencing robot (Applied Biosystems, Waltham, MA, USA) as described by Bensch et al. [30]. The BLAST search tool (National Centre for Biotechnology Information website: http://www.ncbi.nlm.nih.gov/BLAST, accessed on 12 October 2022) was used to determine COLL4 lineage.

### 2.9. DNA Extraction and 16S rRNA Sequencing for Microbiome Analysis

Genomic DNA was isolated from fecal samples of uninfected or *Plasmodium*-infected canaries using a Pure Link Microbiome DNA Purification Kit (Invitrogen, Waltham, MA, USA; Thermo Fisher Scientific, Vallejo St. Emeryville, CA, USA). Final DNA samples were eluted in 70 µL of elution buffer. Genomic DNA quality (OD260/280 between 1.8–2.0) was measured with NanoDrop™ One (Thermo Scientific, Waltham, MA, USA). More than 200 ng of DNA at 20 ng/µL concentration were used for amplicon sequencing of the 16S rRNA gene, which was commissioned to Novogene Bioinformatics Technology Co., (London, UK). Libraries were prepared with NEBNext^®^ Ultra™ IIDNA Library Prep Kit (New England Biolabs, Ipswich, MA, USA). A single lane of Illumina MiSeq system was used to generate 251-base paired-end reads from the V4 variable region of the 16S rRNA gene using barcoded universal primers (515F/806R) in samples from uninfected (*n* = 8) or *P. homocircumflexum*-infected (*n* = 8) canaries. The raw 16S rRNA sequences obtained from bird fecal samples were deposited at the SRA repository (Bioproject No. PRJNA904724).

### 2.10. 16S rRNA Sequences Processing

The software package Quantitative Into Microbial Ecology (QIIME) 2 pipeline (v. 2021.4) [31] was used for the analysis of sequencing data. First, 16S rRNA gene sequences were first demultiplexed and then quality trimmed based on the average quality per base of the forward and reverse reads using DADA2 software [32] implemented in QIIME2. Reads were then merged and chimeric variants were removed. The resulting representative sequences were taxonomically assigned using a pre-trained naïve Bayes taxonomic classifier [33] based on SILVA database version 132 [34] and the 515F/806R primer set. The resulting taxonomic data tables were collapsed at genus level and low abundant taxa were removed by filtering taxa with less than 10 total reads and present in less than 30% of samples. The taxonomic data tables were used for network analysis and keystone taxa identification.

### 2.11. Statistical Analysis

Alpha and beta diversity analyses of taxonomic and functional profiles were performed using rarefied amplicon sequence variants (ASVs) and pathway tables, respectively. Differences in alpha diversity metrics (i.e., Faith’s phylogenetic diversity, observed features and Pielou’s evenness index) within and between different groups were tested using a pairwise Kruskal–Wallis test. Beta diversity was explored using the Bray–Curtis dissimilarity index and compared among the groups using a PERMANOVA test. To test whether factors such as ‘time’ or ‘infection status’ have an impact on beta diversity, an Adonis PERMANOVA test was used. Longitudinal analyses, including comparisons of first differences in alpha diversity metric (i.e., Shannon entropy differences in an individual between different samplings days (DPI)) and first distances (i.e., Bray–Curtis dissimilarity between an individual’s microbiome composition at two separate sampling days), were used to compare the individual’s variation rate over time; specifically, we compared the temporal variations for both metrics in all DPI intervals, using 0 DPI as reference (e.g., form 0 to 8 DPI, from 0 to 16 DPI, and so on). Consequently, the variation rates were compared between uninfected and infected birds using Kruskal–Wallis test and Mann–Whitney U test, respectively. Longitudinal analyses were also performed on the taxonomic profile, detecting and ranking the bacterial genera with the strongest temporal signal (i.e., changes in abundance across time). The longitudinal analyses were performed using the Qiime2 plugin q2-longitudinal pipeline, using the “feature-volatility” function, which detects only features of importance in terms of temporal variations and includes descriptive statistics for all important features. This pipeline mostly uses the random forests ensemble learning as a supervised regression method [35].

Differential abundance of taxa and pathway analyses were performed using the R package ‘DeSeq2′ [36], which applies data normalization based on negative binomial distribution and links variance and mean by local regression, and compares the magnitude of the changes, expressing it as log ratio (log fold change). The comparisons were performed with the Wald test. The number of shared taxa or predicted pathways in the different experimental conditions were visualized using Venn diagrams implemented in the online tool (http://bioinformatics.psb.ugent.be/webtools/Venn/, accessed on 12 October 2022). Differences were considered significant when *p* < 0.05.

For differential network analysis Jaccards index (Jacc) was calculated with Network Construction and Comparison for Microbiome Data (NetComi) script [37] on R studio [38]. This index test for dissimilarities between the “most central nodes” in the networks for degree, betweenness centrality, closeness centrality, eigenvector centrality and for the hub taxa. The “most central nodes” are defined as nodes with a centrality value higher than the empirical 75% quartile. For testing the similarity of most central nodes, two *p*-values *p*(J ≤ j) and *p*(J ≥ j) for each Jacc, which represent the probability that the observed Jacc value is “lower than or equal” or “higher than or equal”, respectively, to the Jacc value expected by random, were calculated taking into account the total number of taxa in both sets [39]. The Jacc ranges from 0 (completely different sets) to 1 (sets equal).

### 2.12. Bacterial Co-Occurrence Networks and Attack Tolerance Test

Co-occurrence microbial networks were built for each condition using the taxonomic profiles at genera level. Microbial networks were used for the graphical representation and visualization of the microbial community assemblies and the quantification of the importance of bacterial taxa in the network community. In the networks, bacterial taxa are represented by nodes and the significant positive (weight > 0.75) or negative (weight < −0.75) co-occurrence interaction between the nodes are represented by edges. The network analysis and construction were performed using Sparse Correlations for Compositional data (SparCC) method [40], implemented in R studio environment [38]. The software Gephi 0.9.5 [41], was used to visualize the microbial networks and to measure the topological features of networks (i.e., number of nodes and edges, network diameter, average degree, weighted degree, average path length, modularity and number of modules). The progression of the ‘microbiome structural state’ was presented using the alpha diversity metric of observed feature [42], measured with QIIME2 pipeline [31], and the number of edges and nodes collected from Gephi [41]. To test the network tolerance to attacks, the Network Strengths and Weaknesses Analysis (NetSwan) script [43] was conducted in R studio [38]. This script simulates a network attack by removing first the nodes with the highest betweenness centrality value, and measures decreases on network connectivity. Connectivity values ranged between 0 (maximum of connectivity between nodes) and 1 (total disconnection between nodes).

### 2.13. Prediction of Functional Traits in the Bird Microbiome

For the metabolic profiling of each sample, PICRUSt2 software [44] was used for the prediction of functional gene abundances based on 16S rRNA gene amplicon sequences. Briefly, the ASVs were aligned and placed into a reference tree (NSTI cut-off value of 2), which was then used to infer gene family copy numbers of each ASVs and finally determine gene family abundance per sample. Kyoto Encyclopedia of Genes and Genomes (KEGG) orthologs (KO) [45], Enzyme Classification numbers (EC) and Cluster of Orthologous Genes (COGs) [46] were used as gene family catalogs for the predictions. Pathway profiles were inferred from structured pathway mapping based on MetaCyc database [47].

## 3. Results

### 3.1. Experimental Infection of Plasmodium homocircumflexum in Birds

**Figure 2:**
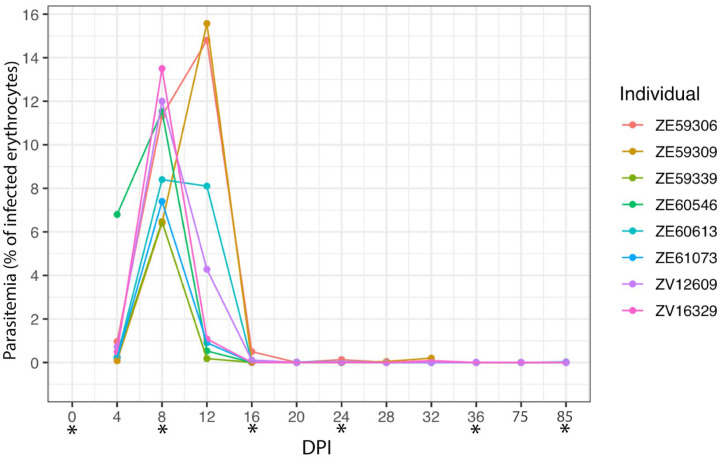
Temporal dynamics of *P. homocircumflexum* parasitemia. Individual parasitemia values (% of infected erythrocytes) of *P. homocircumflexum* based on microscopy are presented. * DPI selected for fecal sample collection and microbiome analysis.

To test how malaria infection modulates the microbiome of vertebrate hosts, we followed an experimental model of avian malaria (Figure 1). All canaries experimentally infected with *P. homocircumflexum* were susceptible to the infection and developed parasitemia (Figure 2). As expected, the control group composed of uninfected birds remained negative to *Plasmodium* infection throughout the experiment. One bird from each group died during the experiment. Based on microscopic examination, the prepatent period, before the parasite appeared in the peripheral blood for the first time, was less than 4 days post-infection (DPI). The dynamics of infection varied individually. The peak of parasitemia was recorded at 8 DPI in six infected birds, and in two canaries the peak was reached at 12 DPI. The parasitemia at the peak varied between 6.5–15.6% of infected erythrocytes. The parasitemia decreased four days after the peak at which point parasitemia was under 0.5% in all infected birds. From 24 DPI onwards the infection fluctuated between disappearance from peripheral blood and very low parasitemia. The parasite was detected in the peripheral blood in two infected birds at 85 DPI.

### 3.2. Changes in the Taxonomic Profiles of Bird Gut Microbiome in Response to P. homocircumflexum Infection

**Figure 3:**
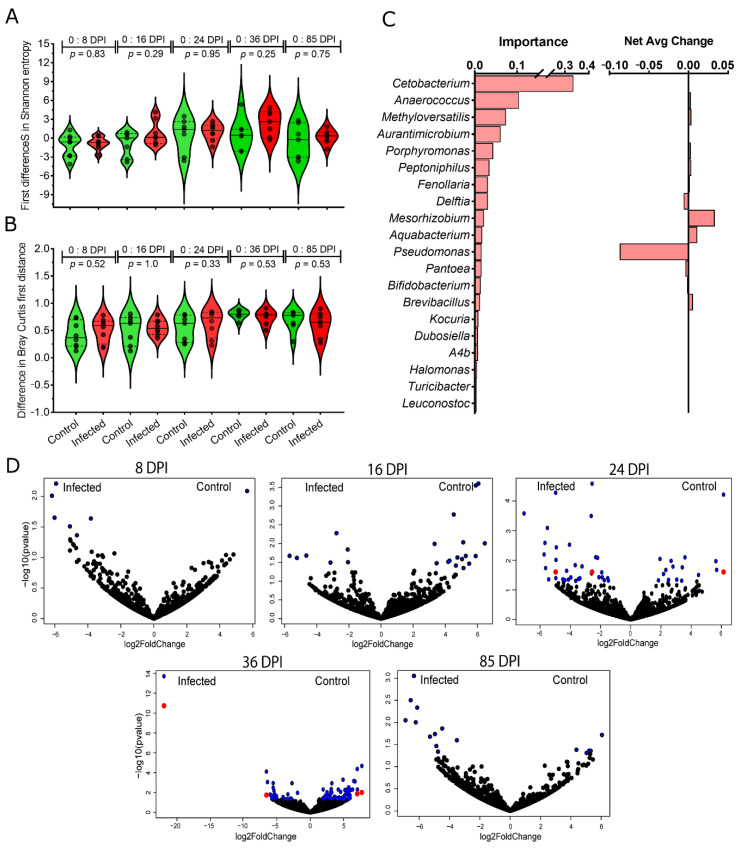
Effect of *P. homocircumflexum* infection on host microbial diversity and taxonomic profiles of bird microbiome. (**A**) Shannon entropy was used in the longitudinal analysis to compare the differences in alpha diversity between infected and uninfected birds at different time intervals (i.e., from 0 to 8 DPI, from 0 to 16 DPI, from 0 to 24 DPI, from 0 to 36 DPI, and from 0 to 85 DPI). Kruskal–Wallis was used to compare the differences (alpha = 0.05). (**B**) Similar longitudinal analysis was performed for beta diversity, based on Bray–Curtis distance. Between-groups comparisons were performed using Mann–Whitney U test (alpha = 0.05). (**C**) Longitudinal feature-volatility analysis of bacterial genera among infected and uninfected birds. The top 20 important features (i.e., from 100 taxa detected by random forest modeling exhibiting important temporal variations in abundance) are shown, including their temporal signal (importance) and net average change. (**D**) Volcano plot showing the differential bacterial abundance in bird microbiome between control and infected groups throughout the duration of experiment. Taxa with significant differences between the groups are represented with blue (Wald test, *p* < 0.05) and red (Wald test, *p* (corrected with Benjamin and Hochberg method) < 0.05) dots. The gray dots represent taxa with no significant differences between groups. Taxa with significant differences in their abundance were identified using DeSeq2 algorithm.

To assess the impact of *P. homocircumflexum* infection on the gut microbiome of canaries, the diversity and composition of amplicon sequence variants (ASVs) of uninfected and *Plasmodium*-infected birds were analyzed in fecal samples collected at different DPI (Figure 1) with variation in parasitemia (Figure 2). Comparison of alpha diversity indexes within groups revealed significant differences in the Faith’s index and observed features (Kruskal–Wallis test: Faith’s index, *p* < 0.001; Observed features, *p* < 0.001), but no in the species evenness (Kruskal–Wallis test, *p* = 0.113). In addition, pairwise comparisons of these alpha diversity indexes between the groups at each sampling day show no significant differences between infected and uninfected birds (Kruskal–Wallis test, *p* > 0.05) (Figure S1). Similarly, beta diversity (Bray–Curtis dissimilarity index) comparisons showed no difference between groups (PERMANOVA, *p* > 0.05), or within groups across time (PERMANOVA, *F* = 1.44; *p* = 0.147) (Figure S2). Further statistical comparison of Bray–Curtis dissimilarity index using multifactorial PERMANOVA (Adonis function), detected significant compositional changes in time, but not associated with the infection state of the birds (Adonis: time, *p* = 0.002; infection, *p* = 0.135, interaction, *p* = 0.285). To further confirm these results, we used the longitudinal statistical approach “q2-longitudinal”, which, by means of machine learning algorithms, analyzes temporal changes in the microbiome. Shannon’s entropy, which combines richness and evenness into one index, showed no significant difference between infected and uninfected birds, regardless of time intervals (Figure 3A). Likewise, no differences in Bray–Curtis’s distance were found for any of the time intervals (Figure 3B). Altogether, these results showed that *P. homocircumflexum* infection does not modify the alpha or beta diversity of bird microbiome.

Taxonomic profiling of microbiome in uninfected and infected birds revealed the presence of a taxonomic core (i.e., taxa identified in all DPI, in either infected or uninfected birds) in both groups, as well as unique taxa associated to different sampling days (Figure S3A). A higher number of unique taxa (125) was found in the taxonomic core of the control group compared to that of the infected group (79), while 268 bacterial genera were present in the taxonomic core of both groups (Figure S3B). The unique taxa found at each sampling day were mostly specific to each group (Figure S3B), and those found only in infected birds were listed (Table S1). This suggested that infection is associated with modulation of the bird microbiome composition.

The longitudinal analysis on the taxonomical profiles revealed a total of 100 “important” taxa in terms of their temporal signal (i.e., their abundance changes gradually over time or are strongly predictive of specific timepoints), considering both infected and uninfected birds (Table S2). The top 20 important taxa included *Cetobacterium* (phylum Fusobacteriota), *Anaerococcus* (Firmicutes), *Methyloversatilis* (Proteobacteria), *Aurantimicrobium* (Actinobacteriota), *Porphyromonas* (Bacteroidota), among others (Figure 3C). Differential analysis of taxa abundance between infected and uninfected birds revealed significant differences only at 24 and 36 DPI in 3 and 4 bacterial genera, respectively, belonging to Proteobacteria (*Escherichia-Shigella*, *Undibacterium*, *Pseudahrensia*, and *Croceicoccus*), Chloroflexi (*Anaerolinea*), Verrucomicrobia (*Opitutaceae*) and Firmicutes (*Lachnospiraceae*) (Wald test, *p* < 0.05; Figure 3D; Table S3). The taxa abundance per phylum shows that the most abundant bacteria in the gut microbiome of birds belongs to Chloroflexi, Proteobacteria, Firmicutes, and Actinobacteriota (Figure S3C).

### 3.3. Changes in Microbial Community Assembly in Response to P. homocircumflexum Infection

**Figure 4:**
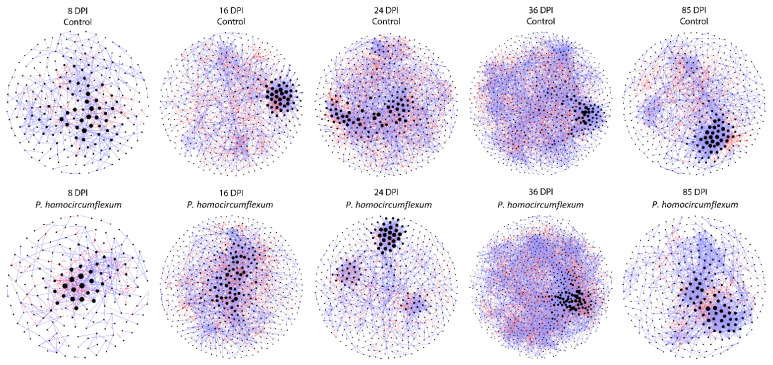
Co-occurrence networks of bird microbiome in the different experimental groups at 8, 16, 24, 36 and 85 DPI. Bacterial co-occurrence networks were inferred from the microbiome of *P. homocircumflexum*-infected birds and control. Nodes represent bacterial taxa and connecting edges stand for a co-occurrence correlation (SparCC > 0.75). Node sizes are proportional to the eigenvector centrality value. Edges representing positive or negative correlations were colored in lilac and red, respectively. Only nodes with at least one connection are displayed.

Bacteria co-occurrence networks were inferred and used to assess the impact of *P. homocircumflexum* infection on the assembly of microbial communities in bird guts at different DPI. Visual inspection revealed that malaria infection changes network topology (Figure 4), and its parameters (Table 1). The difference of topological parameters between *Plasmodium*-infected and control group varied at each sampling day, however more noteworthy differences in topology were recorded at 8 and 24 DPI (Table 1).

**Figure 5:**
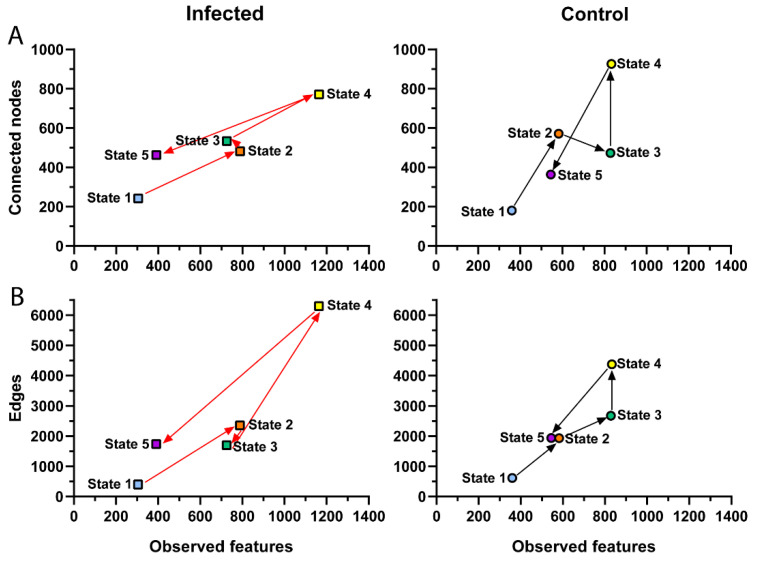
Changes in microbiome structural states of *P. homocircumflexum*-infected and control groups. Scatter plot showing the mean of observed features versus number of (**A**) connected nodes and (**B**) edges found in the microbial co-occurrence networks of infected (**left**) and control (**right**) birds throughout the course of experiment, from state 1 (8 DPI) to 5 (85 DPI) connected by arrows.

To assess the progression of microbiome structural states, we plotted observed features (as a measured of taxa inventory) against connected nodes (as a measure of connectivity), or edges (as a measure of degree) for each DPI in infected and uninfected birds. Thus, ‘observed features-connected nodes’ (Figure 5A) and ‘observed features-edges’ (Figure 5B) plots were referred as ‘microbiome structural states’, from state 1 (8 DPI) to 5 (85 DPI), and the temporal succession of plots (from one DPI to the next) was referred as progression of structural states. The progression of structural states was different in control and *Plasmodium*-infected birds. Notably, transitions from state 2 to state 3 and from state 3 to state 4 were different in infected and control groups. In the microbiome of uninfected birds, the transition from state 2 to state 3 was characterized by an increase in ‘observed features’ with a decrease in ‘connected nodes’ (Figure 5A) and an increase in ‘edges’ (Figure 5B), while in infected birds this transition was characterized by a decrease in ‘observed features’ with an increase in ‘connected nodes’ (Figure 5A) and a decrease in ‘edges’ (Figure 5B). In the control group, the transition from state 3 to state 4 was characterized by no change in ‘observed features’, while in the infected group we observed an increase in ‘observed features’. In both infected and control, transitions from state 3 to state 4 were associated with higher number of ‘connected nodes’ and ‘edges’.

### 3.4. Impact of P. homocircumflexum Infection on Network Centrality Distribution and Robustness

The observed Jacc values for comparisons between networks of infected and uninfected groups were higher than expected by random at 16, 24 and 85 DPI (Table S4). At 8 DPI, the observed Jacc of all centrality measures comparisons was also higher than expected by random, except for betweenness centrality which followed a random distribution (Table S4). At 36 DPI, the observed Jacc of the centrality measures comparisons was higher than expected by random, except for eigenvector centrality and hub taxa, which followed a random distribution (Table S4). It is noteworthy that despite observed Jacc values were higher than expected by random for most centrality measures, these values were lower than 0.5 suggesting low similarity between centrality measures distribution in the microbiome of *Plasmodium*-infected and uninfected birds.

**Figure 6:**
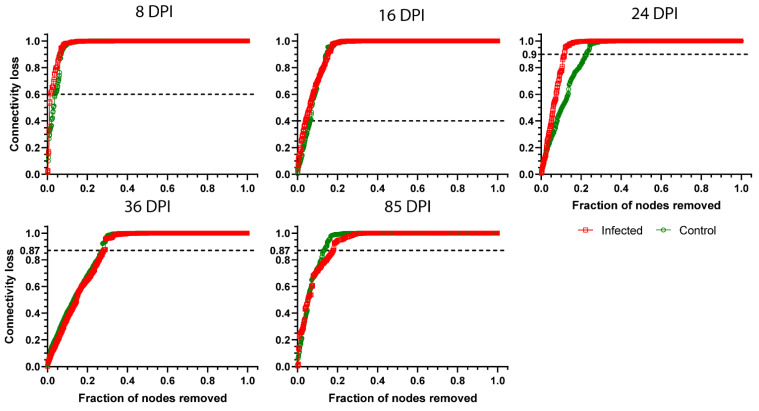
Network tolerance to directed attack. Values of connectivity loss in *P. homocircumflexum*-infected (red squares) and control (green circles) birds at different days of experiment were compared. At each sampling day a threshold (dashed lines) for connectivity loss was determined where the difference of removed nodes between the infected and control group was the largest.

To test whether changes in network structure, and centrality measures impacted the network robustness, we assessed losses in connectivity after directed taxa removal (removing first the nodes with higher betweenness centrality). A major deviation from normal microbiome robustness was observed at 24 DPI in the microbiome of infected birds, while microbiome robustness in the other sampling days was very similar between groups (Figure 6). Particularly, removal of 0.11 and 0.23 fraction of the taxa was enough to reach 90% loss in connectivity in the infected and uninfected groups at 24 DPI, respectively. The results suggest that microbiome modulation due to *Plasmodium* infection reduces network robustness significantly at 24 DPI.

### 3.5. Impact of P. homocircumflexum Infection on the Functional Profiles of Bird Guts Microbiome

**Figure 7:**
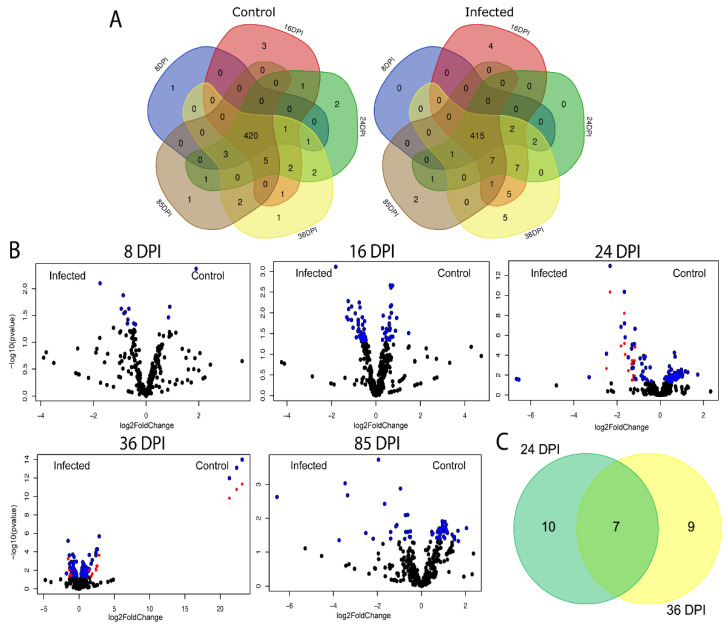
Impact of *P. homocircumflexum* infection on the predicted functional profiles of bird gut microbiome. (**A**) Venn diagram showing the common and different predicted bacterial pathways found in the microbiome of birds infected with *P. homocircumflexum* and uninfected birds at different sampling days. (**B**) Volcano plot showing differential pathway abundance in *Plasmodium-*infected and uninfected birds detected by DESeq2 analysis at 8, 16, 24, 35 and 85 DPI. The blued dots indicate all statistically significant (*p*(un-adjusted) *<* 0.05) pathways, red dots indicate pathways with statistically significant (*p*(adjusted) *<* 0.05) log2 fold changes in the absolute value (cut-off of 1); the black dots are not significant (*p* > 0.05). Detailed information on pathway identity is presented in Table S6. (**C**) Venn diagram. Comparison of unique and shared pathways with significant changes in abundance (*p*(adjusted) < 0.05) between the *Plasmodium*-infected and uninfected groups at 24 and 36 DPI. Only pathways with statistically significant log2 fold changes in the absolute value (cut-off of 1) were considered.

To assess the impact of avian microbiome taxonomic modulation by *P. homocircumflexum* infection on the functional profiles of birds’ microbiome, we performed pathway profiling based on predicted metagenomic functions using PICRUSt2 [44]. The comparison of predicted functional profiles revealed core (i.e., pathways identified in all DPI) and unique pathways in both groups. The functional cores in uninfected and infected birds consisted of 420 (total: 455, 92%) and 415 (total: 455, 91%) pathways, respectively (Figure 7A). Most of the core pathways were shared between the groups (413; total: 455, 91%), with 2 and 7 pathways specific to infected and control group, respectively (Figure S4). Pathways unique to infected birds on 16, 36 and 85 DPI were listed (Table S5).

The comparison of predicted pathway abundance between infected and control birds showed significant fold changes in relative abundance of 17 and 16 pathways at 24 and 36 DPI, respectively (Log2fold change > 1, *p*(adjusted) *<* 0.05, Figure 7B, Table S6). Seven of these were shared between 24 and 36 DPI (Figure 7C). No significant change in predicted pathway abundance was found between groups at 8 DPI, 16 and 85 DPI.

We then tested a possible association between the taxonomic composition and unique pathways present in infected birds at 16, 36 and 85 DPI. The number of taxa contributing to the unique pathways at 16 DPI (33 taxa) and 36 DPI (30 taxa) were similar to each other, but higher to that at 85 DPI (4 taxa) (Figure S5). Most of the contributing taxa were found to be specific to each sampling day. The decreased number of taxa contributing to unique pathways at 85 DPI could be associated with latent stage of *P. homocircumflexum* infection when the parasite impact on bird microbiome is not as prominent as during acute phase.

## 4. Discussion

Natural infection with malaria parasites was associated with changes in the human gut microbiome [48], and the uropygial gland microbiome of house sparrows [11], and experimental infection with murine malaria parasites modulated the gut microbiome of mice [15,16]. We hypothesized that infection with avian malaria would impact bird gut microbiome diversity and structure. To test this hypothesis, we examined microbiome changes caused by experimental infection of *P. homocircumflexum* in canaries. As most available studies on the relation between avian microbiome composition and pathogen encounter focused on economically important species such as chicken and turkey [49], this study is a significant contribution to our understanding of wild bird microbiome in response to infection.

Parasite infection can have synergistic or antagonistic effects on commensal bacteria, effectively shifting microbiome taxonomic [8,50,51] and functional [8] profiles in mammalian [8,15,16] and avian [50,52] hosts. Our results show that microbiome modulation in *Plasmodium-*infected birds was most prominent during the chronic stage of primary infection (from 16 DPI) with a recovered microbiome state by the beginning of the latent stage (85 DPI), although changes of microbiome network topology were observed as early as 8 DPI, at the peak parasitemia. Although here we did not aim at testing the relation between microbiome changes and host health, it is noteworthy that small changes in microbiome composition could affect greatly the health of birds, as fluctuations in microbial functions could have a large impact on microbial-mediated processes such as degradation, detoxification and host defensive mechanisms [3].

We found that alpha diversity of the canary gut microbiome was not greatly affected by *Plasmodium* infection within the tested time points; however, some changes were found in the bacterial composition of *Plasmodium*-infected birds compared with uninfected birds. Similarly, a microbiome study of wild Eurasian tree sparrows by Rohrer et al. [53], found no significant differences in the alpha and beta diversity of birds infected or not with *Plasmodium*. Similarly, Macdonald et al. [52] showed that infection with *Eimeria tenella*, another avian apicomplexan parasite, did not affect microbial alpha diversity of caecal microbiome in chicken within the analyzed time point (i.e., 4 ½ DPI). In contrast, Zhou et al. [50] reported that *E. tenella* infection increased the caecal microbial alpha diversity at 7 DPI. Differences in the tissue tropism of *Plasmodium* (a blood parasite) and *Eimeria* (a prominent gut parasite) in birds may explain differences in their impact on the gut bacterial diversity. Interestingly, infection with murine malaria parasite *P. yoelii* decreased the alpha diversity of the fecal microbiome at 10 DPI, just before a secondary peak of parasitemia, with a gradual recovery by 30 DPI, when the peak parasitemia of *P. yoelii* decreases [15]. Similarly, alpha diversity metrics analysis revealed that infection with *P. berghei* transiently (day 5 to 7) increased richness and evenness, but then these parameters decreased at 9 DPI during acute stage of parasitemia [16]. The results of the latter two studies suggest that murine malaria reduces alpha diversity during acute stage of infection, regardless of parasite species (i.e., *P. yoelii* or *P. berghei*). The results suggest that avian malaria parasites, unlike other apicomplexan pathogens, cause no impact in the bacterial diversity of infected birds.

In this study, avian *Plasmodium* infection had the highest impact on the abundance of genera of the phyla Proteobacteria (*Escherichia-Shigella*, *Undibacterium*, *Pseudahrensia*, and *Croceicoccus*), followed by Chloroflexi (*Anaerolinea*), Verrucomicrobia (*Opitutaceae*) and Firmicutes (*Lachnospiraceae*). Specifically, infection increased and decreased the abundance of Proteobacteria taxa in the infected birds at 24 and 36 days, respectively. *Plasmodium berghei* infection reduced Firmicutes in two lines of laboratory mouse, C57BL/6 and BALB/c, and increased Proteobacteria and Verrucomicrobia in C57BL/6 mice over a period of 9 days during acute stage of infection [16]. Fecal microbiome of *P. yoelii*-infected C57BL/6 mice showed a decreased abundance of Firmicutes and a relative increase in the abundance of Bacteroidetes at the peak stage of infection, which reverted to baseline by 30 days when parasitenia decreased [15]. Similarly, *P. yoelii* infection induced a reduction in the abundance of Firmicutes and an increase in the abundance of Bacteroidetes in the gut microbiome of BALB/c mice [54]. In addition, changes to the intestinal milieu caused by *P. yoelii* infection promote colonization of the intestine with both *Salmonella enterica* serotype Typhimurium and *Escherichia coli* [15]. In case of avian malaria, the changes in bacteria abundance appeared at a chronic stage of infection concurring with low parasitemia, while rodent malaria effects were detected when the parasite was still at the primary stage of development. Our results together with previously published studies on murine malaria [15,55] suggest that the impact of malaria infection on microbiome is mainly transient and can affect different bacterial taxa.

Bacterial communities are dynamic systems with high intra-individual compositional variability across time [55,56], and infection causes deviations from normal microbiome dynamics across biological systems, from animals [57] to plants [58,59]. Similarly, infection of mice with the pathogen *Citrobacter rodentium* revealed unique, time-dependent microbial signatures associated with host response to infection [57]. Compositional changes may impact community assembly and co-occurrence networks [60]. We found that network topology and progression of microbiome structural states were affected by infection. Particularly, we found that arrival to, and departure from, state three (24 DPI) was different in infected birds compared with the control group.

Notably, network of infected birds in state three were less robust than the control network. These results suggest that ‘state three’ plays a key role on the temporal dynamics of bird microbiome. Network robustness, measured here as tolerance to sequential removal of highly-central nodes [61], is a lower-order connectivity feature investigated at the level of individual nodes and edges [62,63,64]. There is a possibility that the accumulation of some taxa facilitated by infection and/or removal of other taxa excluded by infection within the first 24 DPI change microbe-microbe interactions affecting the hierarchical organization (who influences who) of the community. Resulting redistribution of betweenness centrality values in the network could in turn affect network robustness; with networks having higher betweenness centrality nodes tending to be more vulnerable to directed attacks [64]. Although the Jacc for betweenness centrality at 24 DPI was higher than expected by random, the Jacc value was lower than 0.50 suggesting low similarity in the distribution of betweenness centrality values among nodes in infected and uninfected networks. The combination of Jacc and robustness analysis supports that redistribution of betweenness centrality values induced by infection at 24 DPI reduces network robustness at 24 DPI. In addition to lower-order connectivity features, higher-order network features (i.e., patterns of interconnections or network motifs [65]), play a fundamental role in understanding the organization of many complex systems [65], and affect network robustness [64,66,67]. Occurrence of motifs in complex networks is not random, as motifs tend to perform important functions [68,69]. Thus, robustness reduction in state three networks of infected birds could be associated not only with redistribution of betweenness centrality across nodes, but also with modifications in patterns of network motifs. Further studies should address whether there is an association between network motifs and network robustness in the avian malaria system presented here.

Despite it is not clear how malaria parasites interact with intestinal microbiota to induce dysbiosis [13,14], several mechanisms can potentially account for changes in bacterial abundance and composition caused by *Plasmodium* infection [12,14]. The host-parasite relationships during malarial infections include immune response of the host that may directly, or indirectly, impact the host microbiome composition during malaria infections [14]. For example, activation and degranulation of mast cells during human and murine malaria damage the gastrointestinal barrier resulting in release of intestinal bacteria into the bloodstream [70]. Concomitantly, hemolysis caused by *Plasmodium* infection of red blood cells decreases reactive oxygen species (ROS) production by neutrophils increasing the growth of intestinal invading bacteria within these polymorphonuclear leukocytes [71]. The occurrence of some of these potential mechanisms is yet to be tested in birds.

Beyond changes in taxonomic profiles, the metabolites produced by specific bacterial species in the gut exert systemic effects on the host [72], as well as influence malaria infection and disease severity [73]. According to our study, most of the unique pathways identified in the microbiome of *Plasmodium*-infected birds could not be directly associated with avian malaria infection, however a PWY5F9-12 (biphenyl degradation) and PWY-3081 (L-lysine biosynthesis V) could be involved in anti-malarial response [74,75]. The presence of pathways of toluene degradation in the *Plasmodium*-infected birds could also be as response to the infection due to the production of toluene by the parasite [76,77].

## 5. Conclusions

Microbiota diversity in wild birds is highly influenced by the host ecology. As a consequence, microbiome composition and assembly may be under selective pressures by parasites in natural systems. We found that infection with the avian malaria parasite *P. homocircumflexum* modulated the fecal microbiome of canary hosts causing deviation from normal development, while no significant change in bacterial diversity was observed. The results showed changes in community assembly as early as 8 DPI, followed by more prominent fluctuations in bacterial composition with the emergence of infection-specific taxa and pathways at the later stages of infection. Although the microbiome of *Plasmodium*-infected birds has mainly recovered by 85 DPI, latent malaria infection could have long-lasting effect in the host microbiome with impact on bird health [3], and potentially host evolution. In addition, the spread of low virulent avian malaria parasites across a bird population could select for individuals carrying an infection-permissive microbiome. Parasite infection may select against the occurrence or high abundance of bacteria associated with anti-malarial mechanisms. For example, bacteria within the family Enterobacteriaceae are a rich source of α1,3-galactosyltransferase (α1,3GT) activity. Immune response to the carbohydrate α-Gal on the surface of microbiota bacteria in birds could trigger anti-α-Gal antibodies [7], with malaria killing activity [78]. Interestingly, the abundance of *Escherichia-Shigella* was significantly reduced in *P. homocircumflecxum*-infected birds at 24 DPI. By reducing the occurrence of Enterobacteriaceae across the bird population the parasite may reduce anti-malarial immunity at the population level.

## Figures and Tables

**Table 1:** Topological parameters of co-occurrence networks.

Network Features	8 DPI *		16 DPI		24 DPI		36 DPI		85 DPI	
	Control	*Plasmodium*-Infected	Control	*Plasmodium*-Infected	Control	*Plasmodium*-Infected	Control	*Plasmodium*-Infected	Control	*Plasmodium*-Infected
Nodes	242 (688) **	180 (641)	482 (938)	571 (1100)	534 (938)	473 (1023)	771 (1059)	926 (1267)	463 (926)	364 (666)
Edges	617	399	1929	2354	2673	1597	4375	6299	1934	1736
Positive	453 (73.4%)	245 (61.4%)	1130 (58.6%)	1467 (62.3%)	1597 (59.8%)	1079 (67.6%)	2877 (65.8%)	4169 (66.2%)	1423 (73.6%)	1403 (80.8%)
Negative	164 (26.6%)	154 (38.6%)	799 (41.4%)	887 (37.7%)	1076 (40.2%)	518 (32.4%)	1498 (34.2%)	2130 (33.8%)	511 (26.4%)	333 (19.2%)
Network diameter	15	16	11	13	9	12	8	13	12	17
Average degree	5.099	4.433	8.004	8.245	10.011	6.753	11.349	13.605	8.354	9.538
Weighted degree	1.928	0.827	1.124	1.67	1.651	1.946	2.897	3.649	3.254	4.84
Average path length	4.434	5.367	4.551	4.279	3.914	5.046	3.72	3.985	4.321	4.944
Modularity	1.13	2.118	2.85	2.001	2.345	1.474	1.657	1.543	1.013	0.939
Number of modules	55	49	75	106	59	84	63	61	73	45
Average clustering coefficient	0.43	0.422	0.529	0.459	0.466	0.448	0.475	0.446	0.458	0.473

* DPI—days post-infection. ** a number of nodes with at least one connection (a total number of nodes).

## Data Availability

The datasets presented in this study can be found in SRA repository (Bioproject No. PRJNA904724).

## Supplementary Materials

The following supporting information can be downloaded at: https://www.mdpi.com/article/10.3390/microorganisms11030563/s1, Figure S1: Alpha diversity indexes of infected and control bird microbiome. (A) Faith’s phylogenetic diversity index, (B) observed features, (C) Pielou’s evenness index, were calculated at the level of ASVs and compared between infected and control groups for each DPI. Control birds, *n* = 8; infected birds, *n* = 8. Days post-infection (DPI); Figure S2: Beta diversity of infected and control bird microbiome. (A) Bray–Curtis distance from each time point is represented in PCoA plot obtained by Betadisper function. (B) Bray–Curtis volatility plot, indicating how the microbiome composition changed in each group over time. The lines represent the mean and the standard error mean from each group. Control birds, *n* = 8; infected birds, *n* = 8. Days post-infection (DPI); Figure S3: Shared and unique bacterial genera found in the microbiome of infected and uninfected birds. (A) Venn diagrams displaying the number of shared and unique taxa detected within groups at different time points. (B) Unique taxa detected at the different time points in uninfected and infected birds were compared and displayed in Venn diagrams; the percentage at the sides of the core, shows proportional size of shared taxa in infected and control groups. (C) Taxa abundance per phylum in the microbiome of infected and control birds at different time points of experiment. Control birds, *n* = 8; infected birds, *n* = 8. Days post-infection (DPI); Figure S4: Shared and unique metabolic pathways found in the microbiome of infected and uninfected birds. Unique pathways of infected/control groups at different experimental days were selected and compared. The functional profiles of microbiome were predicted from 16S rRNA amplicon sequences using PICRUSt2. Control birds, *n* = 8; infected birds, *n* = 8. Days post-infection (DPI); Figure S5: Taxa contribution to metabolic pathways. Bacterial taxa contribution to unique pathways in the gut microbiome of infected and control birds at 16, 36 and 85 DPI is displayed as Sankey diagrams. Taxonomic contribution was assessed using PICRUSt2. Node size is proportional to the abundance of contributing taxa or pathways. The contribution of each taxon to different pathways is represented proportionally by the size of cords. Days post-infection (DPI); Table S1: List of unique bacterial taxa in *P. homocircumflexum*-infected birds at different DPI and taxonomic core (i.e., taxa identified in all DPI) are listed; Table S2: List of taxa with important changes in abundance across time; Table S3: List of differentially abundant taxa identified by the DESeq2 method between the gut microbiome of *P. homocircumflexum*-infected and uninfected birds at 24 and 36 DPI. Only taxa with statistically significant log2 fold changes in the absolute value (cut-off of 1) are presented (*p*(adjusted) < 0.05); Table S4: Jaccard indexes of local centrality measures. Jaccard’s indexes for each of local centrality measures (i.e., degree, betweenness centrality, closeness centrality, eigenvector centrality and hub taxa) of the sets of most central nodes for pairwise network comparisons. The two *p*-values, *p*(J ≤ j) and *p*(J ≥ j), for each Jaccard’s index were added. * *p* < 0.05, ** *p* < 0.01; *** *p* < 0.001; Table S5: List of pathways unique to *P. homocircumflexum*-infected birds on 16, 36 and 85 DPI; Table S6: List of differential pathways identified by the DESeq2 method between the gut microbiome of *P. homocircumflexum*-infected and uninfected birds at 24 and 36 DPI. Only pathways with statistically significant (*p*(adjusted) < 0.05) log2 fold changes in the absolute value (cut-off of one) are listed.
