# Supplementary material for: Avian Malaria Parasites Modulate Gut Microbiome Assembly in Canaries
Source: Microorganisms. 2023 Feb 23;11(3):563. doi: 10.3390/microorganisms11030563 (PMC10056159; doi:10.3390/microorganisms11030563)
Supplement: Supplementary file 1 [file microorganisms-11-00563-s001.zip › Supplementary Table S3.pdf]

Supplementary Table S3. Bacterial taxa with changes in abundance.

Time points	Bacterial genera	Bacterial phylum	Note	Wald test, *p* value
24 dpi	g__*Escherichia-Shigella*	Proteobacteria	More abundant in uninfected birds	$p < 0.05$
	g__*Undibacterium*	Proteobacteria	More abundant in infected birds	$p < 0.05$
	g__*Anaerolinea*	Chloroflexi	More abundant in uninfected birds	$p < 0.05$
36 dpi	g__*Pseudahrensia*	Proteobacteria	More abundant in uninfected birds	$p < 0.05$
	f__*Opitutaceae* (uncultured)	Verrucomicrobia	More abundant in infected birds	$p < 0.05$
	g__*Croceicoccus*	Proteobacteria	More abundant in infected birds	$p < 0.05$
	f__*Lachnospiraceae* (CAG-56)	Firmicutes	More abundant in uninfected birds	$p < 0.05$
