# Supplementary figures and images for: Avian Malaria Parasites Modulate Gut Microbiome Assembly in Canaries
Source: Microorganisms. 2023 Feb 23;11(3):563. doi: 10.3390/microorganisms11030563 (PMC10056159; doi:10.3390/microorganisms11030563)

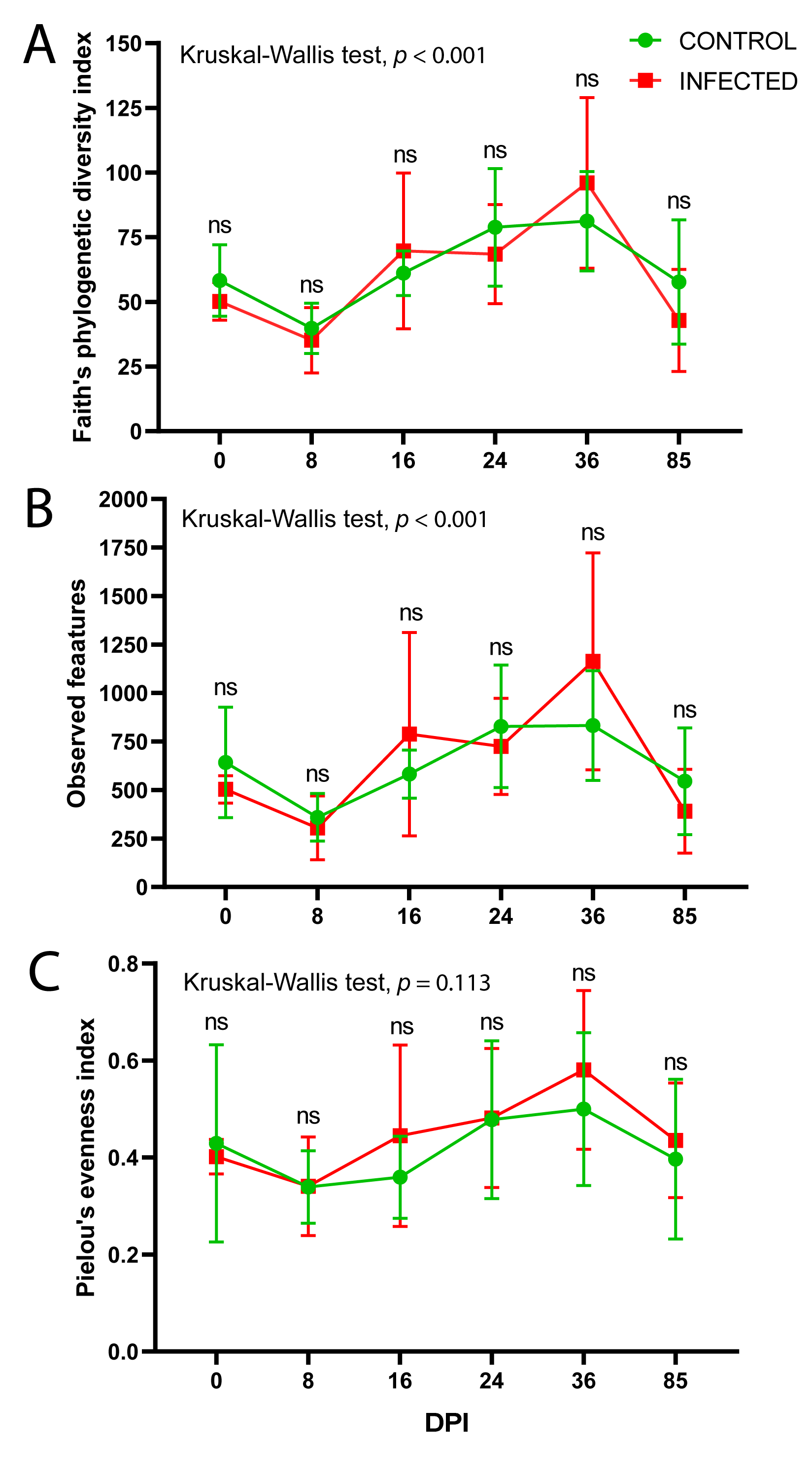

Supplement: Supplementary file 1 [file microorganisms-11-00563-s001.zip › Supplementary Figure S1.tif]

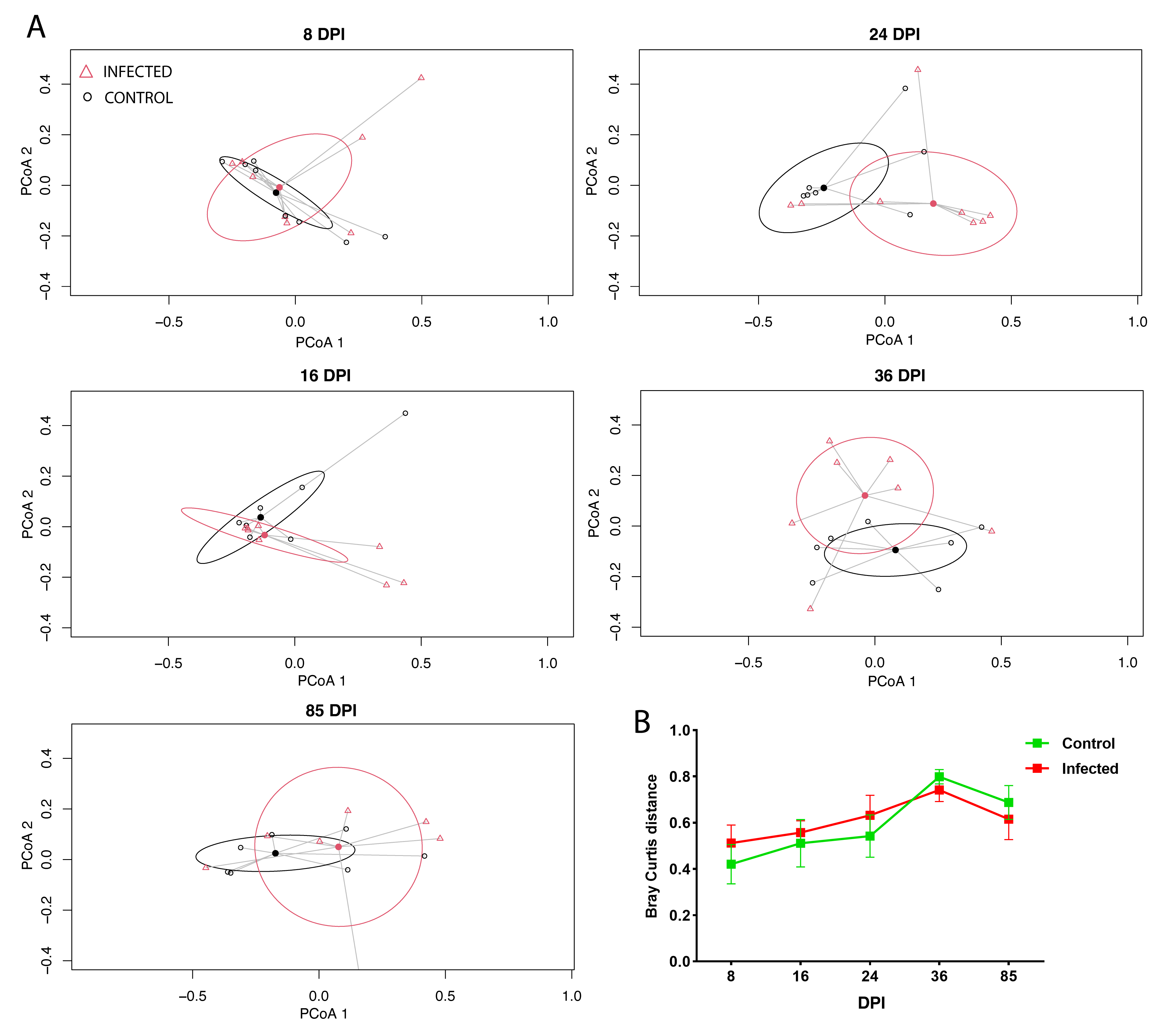

Supplement: Supplementary file 1 [file microorganisms-11-00563-s001.zip › Supplementary Figure S2.tif]

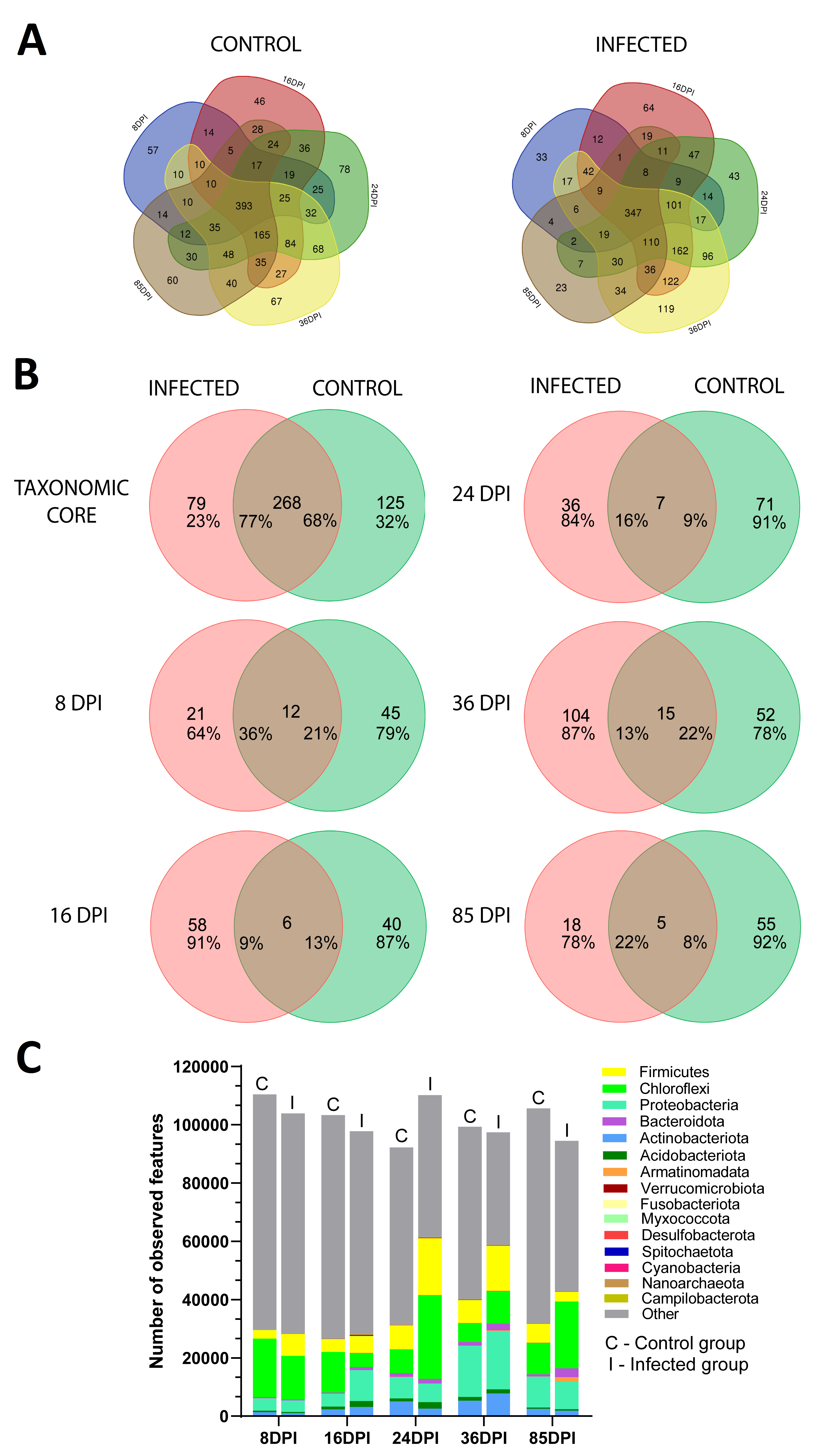

Supplement: Supplementary file 1 [file microorganisms-11-00563-s001.zip › Supplementary Figure S3.tif]

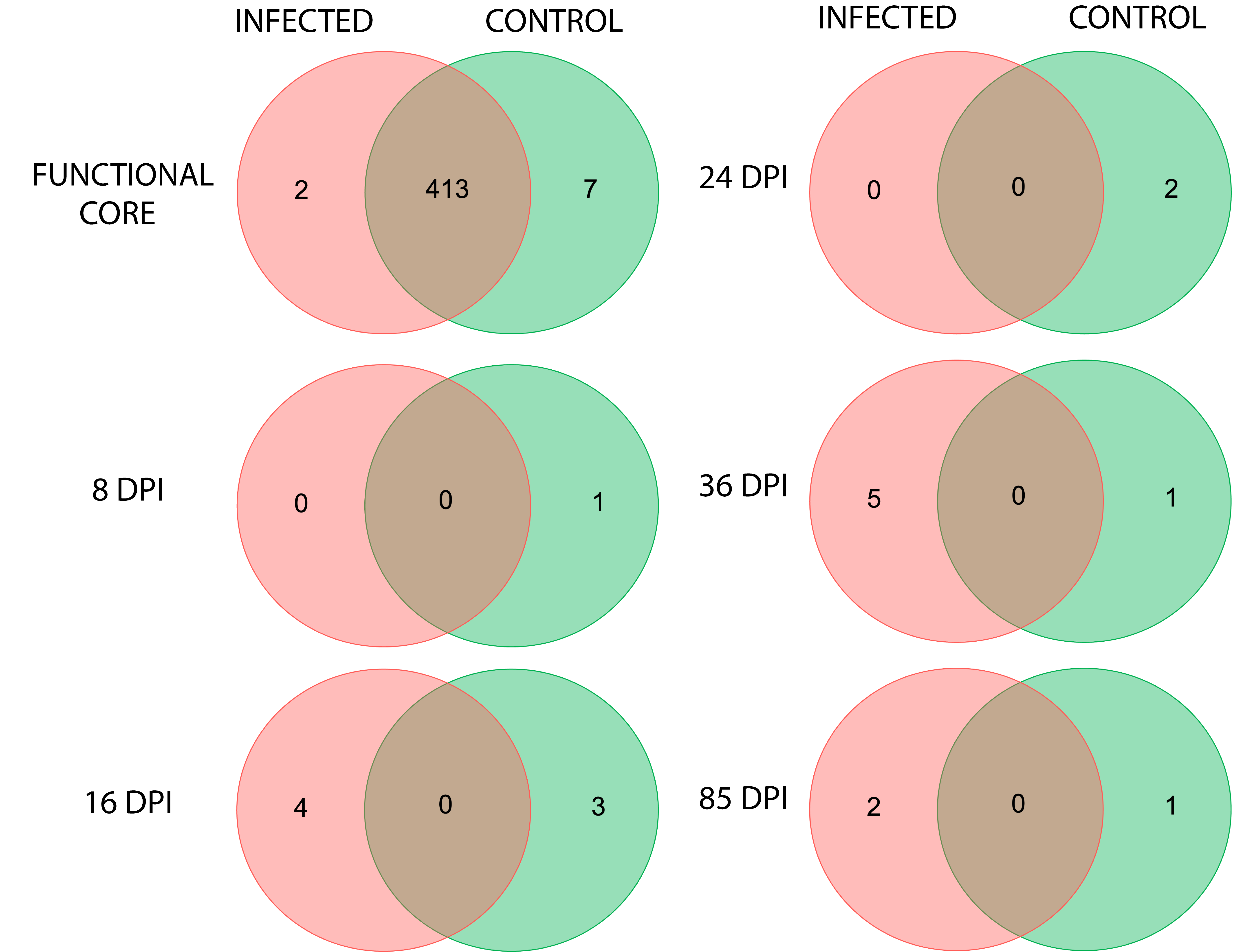

Supplement: Supplementary file 1 [file microorganisms-11-00563-s001.zip › Supplementary Figure S4.tif]

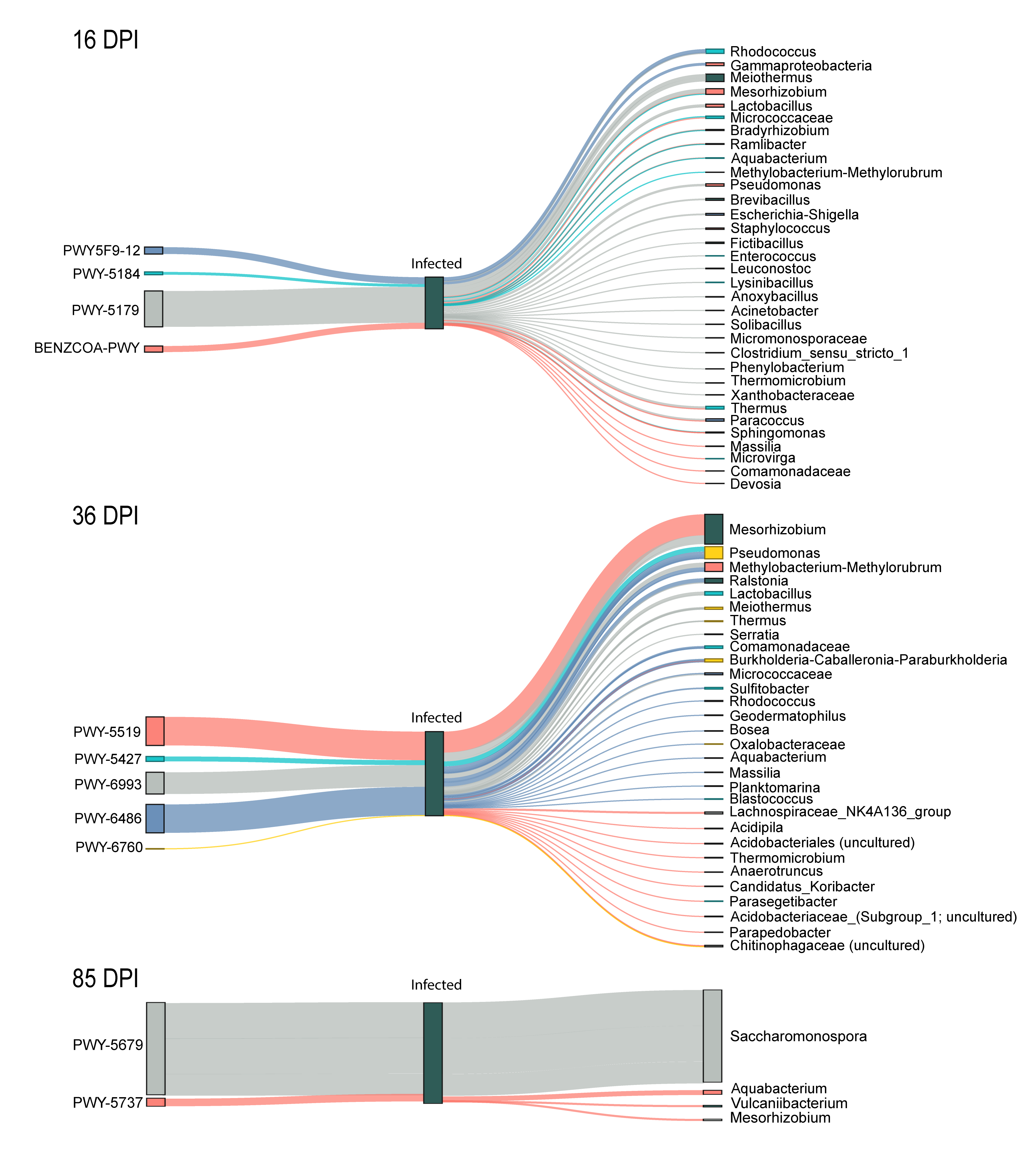

Supplement: Supplementary file 1 [file microorganisms-11-00563-s001.zip › Supplementary Figure S5.tif]
